# Predictors of middle school students’ perceptions of automated writing evaluation^☆^

**DOI:** 10.1016/j.compedu.2023.104985

**Published:** 2024-04

**Authors:** Joshua Wilson, Fan Zhang, Corey Palermo, Tania Cruz Cordero, Matthew C. Myers, Halley Eacker, Andrew Potter, Jessica Coles

**Affiliations:** a School of Education, University of Delaware, United States; b Measurement Incorporated, United States

**Keywords:** Automated writing evaluation, Automated feedback, Writing, Social validity, Perceptions

## Abstract

This study examined middle school students' perceptions of an automated writing evaluation (AWE) system, *MI Write*. We summarize students' perceptions of MI Write's usability, usefulness, and desirability both quantitatively and qualitatively. We then estimate hierarchical entry regression models that account for district context, classroom climate, demographic factors (i.e., gender, special education status, limited English proficiency status, socioeconomic status, grade), students' writing-related beliefs and affect, and students' writing proficiency as predictors of students' perceptions. Controlling for districts, students reporting more optimal classroom climate also reported higher usability, usefulness, and desirability for MI Write. Also, model results revealed that eighth graders, students with limited English proficiency, and students of lower socioeconomic status perceived MI Write relatively more useable; students with lower socioeconomic status also perceived MI Write relatively more useful and desirable. Students who liked writing more and more strongly believed that writing is a recursive process viewed MI Write as more useable, useful, and desirable. Students with greater writing proficiency viewed MI Write as less useable and useful; writing proficiency was not related to desirability perceptions. We conclude with a discussion of implications and future directions.

## Highlights

• We study middle school students' perceptions of an AWE system called MI Write.
• Students with LEP and lower SES perceived MI Write more useable/useful.
• So too did students who liked writing and believed that revising was important.
• Less proficient writers more strongly agreed that MI Write was useable and useful.

## 1 Introduction

Writing is a goal-directed problem-solving process that involves planning, translating, transcribing, and reviewing/revising (Hayes, 1996). Thus, writing is not just the physical act of putting words on paper or typing on a keyboard but also involves the writer's ability to engage in self-directed cognitive, metacognitive, and affective processes. Learning to write involves the maturation of those processes via the influence of various social contexts, including formal education (Graham, 2018), as well as deliberate practice guided by feedback from teachers, peers, oneself, and technology-based writing programs (Graham et al., 2015, Palermo & Wilson, 2020).

Writing plays an essential part in academic success, as writing is not only an academic outcome in and of itself, but also an important tool for learning and deepening understanding of course content (Bangert-Drowns et al., 2004; Graham & Perin, 2007). However, most students in the United States do not attain grade-level writing proficiency (National Center for Education Statistics, 2012). Therefore, it is imperative to develop innovative, effective, and engaging instructional methods to improve students’ writing, specifically their ability to compose well-organized and elaborated texts that demonstrate a command of written language (e.g., sentence fluency and vocabulary) and its conventions (i.e., spelling, punctuation, grammar) in order to achieve a variety of communicative purposes (National Assessment Governing Board, 2017).

In recent years, technology has profoundly transformed language education, particularly in the realm of writing instruction (Wen & Walters, 2022), and specifically via the use of automated writing evaluation (AWE; Fleckenstein et al., 2023; Ngo et al., 2022; Nunes et al., 2022). Studies employing activity theory and examining AWE-supported writing processes have shed light on technology's impact on educational contexts (Chen et al., 2022; Li, 2022). This evolution is evident particularly in second language (L2) learning contextx, where technology-mediated writing processes have gained prominence (Han et al., 2021; Loncar et al., 2023; Rohayati & Kosasih, 2023). Results of both meta-analyses (e.g., Vitta & Al-Hoorie, 2020) and individual studies (e.g., Zhang & Zhang, 2018) show technology's transformative potential for engaging learners and enhancing writing skills. Results collectively underscore the dynamic nature of language education, where technology serves as a potent scaffold for shaping writing processes and language skill acquisition.

Based on recent meta-analyses (Fleckenstein et al., 2023; Graham et al., 2015; Li, 2022; Zhai & Ma, 2022b), one promising technology-based intervention is AWE. AWE is software that uses natural language processing to provide immediate, computer-generated evaluative scores and feedback (Hockly, 2019; Strobl et al., 2019). Modern day AWE systems also have other features, such as peer review, teacher reporting functions, and embedded skill-building opportunities. In so doing, AWE can accelerate the practice–feedback loop necessary for writing development (Kellogg et al., 2010).

AWE capabilities have expanded in recent years (Deeva et al., 2021; Huang et al., 2023), accompanied by increased AWE adoption in schools. Along with research evaluating the effectiveness of AWE, which shows generally positive findings (Ersanli & Yesilel, 2023; Fleckenstein et al., 2023; Li, 2022; Zhai & Ma, 2022b), it also is important that research evaluate stakeholders' perceptions of the *social validity* of AWE (see Shi & Aryadoust, 2022). Social validity refers to how the goals, procedures, and effects of an intervention are perceived by its recipients and/or implementers (Kazdin, 1977; Wolf, 1978) and may also include perceptions of an intervention's usability, usefulness, and desirability. In this study, we define *perceptions* as beliefs and opinions held by stakeholders following their interactions with AWE. Usually, social validity is measured through self-report, interview, or focus group methods (Common & Lane, 2017).

Results of several studies underscore the significance of a multifaceted investigation into students' acceptance of AWE feedback. For example, by employing the Technology Acceptance Model to examine college students' acceptance of AWE, Zhai and Ma (2022a) reported that perceived ease of use (i.e., usability) and perceived usefulness both positively predict students' acceptance of AWE; however, college students place greater importance on perceived usefulness over perceived usability. Similarly, relevant research has operationalized social validity through the three dimensions of usability, usefulness, and desirability, demonstrating the value of assessing each of these dimensions to understand users' perceptions and experiences with educational technologies (Palermo & Wilson, 2020; Lyst et al., 2005; Roscoe et al., 2018; Wang et al., 2020; Wolf, 1978). We incorporate these dimensions in the present study given the need for a holistic evaluation of AWE's social validity, aligned with students' needs and preferences.

When the social validity of AWE is considered from teachers’ perspectives, research indicates that teachers generally report positive perceptions of AWE (Correnti et al., 2022; Koltovskaia, 2022; Li et al., 2015, Wilson et al., 2021, Wilson & Czik, 2016, Wilson, Huang, Palermo, Beard, & MacArthur, 2021). Both elementary school teachers (Wilson, Ahrendt et al., 2021) and middle school teachers (Grimes and Warschauer, 2010, Palermo & Thomson, 2018, Wilson & Czik, 2016) indicate that AWE saves them time, makes writing instruction easier, and lets them focus their own feedback on higher-level concerns instead of mechanics.

However, teachers also indicate that AWE creates new instructional challenges, which factor into their social validity perceptions. For instance, the same elementary teachers who praised AWE also commented that using AWE is challenging because it applies feedback very differently than they do. Specifically, while teachers' feedback accounts for student effort and timing of skill acquisition across a school year, automated feedback ignores those factors, causing teachers to perceive automated feedback as less personalized and more standardized, and, in some cases, to hold unrealistic expectations for students' performance (Wilson, Ahrendt et al., 2021). Even when AWE feedback is deemed to align with instructional goals, teachers indicate that not all students understand or benefit from automated feedback, and still require teacher support (Correnti et al., 2022). At the post-secondary level, teachers report that despite its benefits, AWE is less accurate at detecting student errors, which undermines teachers’ trust in the technology (Chen & Cheng, 2008; Jiang & Yu, 2020).

Less research has evaluated students' perceptions of AWE, especially L1 and K–12 students (versus L2 and postsecondary students; Fu et al., 2022). However, it is critical to understand students' perspectives on AWE, as these perceptions profoundly influence their engagement, motivation, and overall experience with AWE (Brine & Franken, 2006; Cotos et al., 2017; Rohayati & Kosasih, 2023), as well as their intention to use AWE in the future (Zhai & Ma, 2022a). Particularly lacking is research seeking to understand what factors—be they contextual, demographic, affective, or cognitive—predict students’ perceptions of AWE, and the extent to which those factors vary over time. Once identified, such factors can aid researchers, practitioners, and AWE developers in identifying student populations that are more likely to perceive AWE as beneficial and those that may need additional support to do so.

### 1.1 Students’ perceptions of AWE

On average, students hold positive perceptions of AWE, finding it to be beneficial to the writing process and learning to write. However, students' perceptions of AWE appear to vary depending on students’ educational level. Postsecondary students typically report mixed and critical attitudes toward AWE (e.g., Li et al., 2017; Link et al., 2014; Link et al., 2022; Lu et al., 2015; Ranalli, 2018; Scharber et al., 2008), whereas K–12 students typically report more favorable perceptions (Grimes and Warschauer, 2010, Klobucar et al., 2013, Palermo & Thomson, 2018, Palermo & Wilson, 2020, Raković et al., 2021, Ware, 2014, Wilson, Huang, Palermo, Beard, & MacArthur, 2021, Wilson & Roscoe, 2020).

Also, students' perceptions of AWE appear to vary depending on students' writing proficiency. For example, in a study exploring Chinese postsecondary students' perceptions of *Pigai*—the largest and most popular AWE in China—less proficient participants evaluated the comprehensibility and usefulness of Pigai's automated feedback more positively (Huang & Renandya, 2020). Such findings have led some researchers to conclude that the current capabilities of AWE may be better suited to meet the needs of students who are less proficient writers (e.g., Ranalli et al., 2017).

In contrast, more proficient writers have criticized AWE for inaccuracies in error detection and for its generic feedback (Fu et al., 2022), preferring instead to receive feedback from an instructor or a peer (Bai & Hu, 2017; Chen & Cheng, 2008; Gao, 2021). Indeed, AWE systems are less precise than instructors at classifying grammatical errors and identify fewer errors overall (i.e., less ‘recall’) (Shadiev & Feng, 2023), which can undermine students' trust and positive regard for AWE (e.g., Bai & Hu, 2017; Chen & Cheng, 2008, c. f., Dikli & Bleyle, 2014). Indeed, students' appraisal of AWE's scoring accuracy and feedback accuracy relates to their social validity perceptions (Roscoe et al., 2018, Roscoe, Wilson, Johnson, & Mayra, 2017).

Importantly, even among younger and less proficient writers, students can distinguish between how easy AWE is to use (i.e., its usability), how beneficial it is (i.e., its usefulness), and how much they wish to use AWE again or would recommend it to other students (i.e., its desirability). For example, Wilson, Huang et al. (2021) asked students in Grades 3–5 to rate the usability, usefulness, and desirability of an AWE system called *MI Write* after a year of districtwide implementation of the software. Students reported the highest agreement regarding MI Write's usability and certain aspects of usefulness, namely whether MI Write helped them identify areas of improvement and revise their writing. Students reported the lowest agreement regarding MI Write's usefulness for improving their writing motivation and its desirability.

In sum, prior research indicates that students tend to hold positive perceptions of AWE, but older and more skilled writers tend to be more critical. Nonetheless, students appear able to evaluate discrete aspects of social validity, including usability, usefulness, and desirability.

### 1.2 Present study

Additional research is needed to better understand students’ perceptions regarding the social validity of AWE and the factors that predict these perceptions. Such findings can assist researchers, practitioners, and AWE developers in identifying student populations that are prone to positively perceive AWE as beneficial, as well as populations that may require additional implementation support. Crucially, identifying these populations can aid in planning AWE implementations and interventions, with potential implications for differentiation of supports, and can inform AWE developers in expanding design considerations to encompass a wider range of students, thereby promoting greater inclusivity.

To address these research gaps, we examined perceptions of a diverse sample of US middle school students who used MI Write in the context of core English language arts (ELA) instruction during the 2021–2022 school year. Our primary goal was to identify factors that explained the variability in students’ perceptions and to investigate any changes in these factors following the implementation of MI Write.

In our investigation, we considered various factors that could potentially influence students' perceptions of AWE. First, we examined variables that accounted for the district and classroom context in which MI Write was implemented, as prior research has shown that AWE implementation can vary across districts and classrooms (Deane et al., 2021; Mayfield and Butler, 2018, Wilson, Myers, & Potter, 2022) and such variation is reasonably related to students’ perceptions of AWE.

Given prior research showing that demographic factors appear to be associated with students' perceptions of AWE, we also examined students' gender, special education status, limited English proficiency status, socio-economic status, and grade level. Some or all these demographic factors may be associated with students’ perceptions of AWE because they have been linked to differences in writing proficiency and writing motivation (Deane, 2023) and access to educational technology (Lu & Overbaugh, 2009; Warschauer et al., 2004).

We also considered factors pertaining to students' writing-related beliefs and affect towards writing. Specifically, we considered students' writing self-efficacy, liking of writing, and the degree to which they believed that writing is a process requiring revision (i.e., *recursive process beliefs*). Differences in writing-related beliefs and affect likely influence how students engage with feedback (Sherf & Morrison, 2020; Winstone et al., 2021), including automated feedback. Thus, such factors may reasonably be associated with students’ perceptions of AWE.

Finally, given prior research showing an association between students’ level of writing proficiency and their perceptions of AWE (Fu et al., 2022; Ranalli et al., 2017), we also considered this factor in our investigation. Students with different levels of writing proficiency may have different expectations, preferences, and needs when utilizing AWE.

Accordingly, the following research questions guided our study:

RQ1 What are middle-school students' perceptions of AWE's usability, usefulness, and desirability?

Based on prior research, we hypothesized that middle school students’ perceptions generally would be positive but would differ across these three aspects.

RQ2 What factors predict students' perceptions of AWE's usability, usefulness, and desirability? To what extent do these factors change following the implementation of AWE?

It is crucial to identify factors that predict which students are likely to perceive the benefits of AWE before and after using it. By understanding these factors, stakeholders can better design implementation supports to reach a broader audience of students, including those who may not immediately see the benefits of AWE or who may lack motivation to use it. This knowledge is essential to the effective implementation and expansion of AWE in educational settings, as it can help ensure that students who would most benefit from the technology receive it and are motivated to use it. We predicted that writing proficiency would be associated with students’ perceptions of AWE; however, given the dearth of research in this area, we held no other strong a priori hypotheses about which specific variables would prove most influential in predicting students' perceptions of AWE.

## 2 Methods

### 2.1 Sample

We collected data from treatment students (*N* = 1299) in Grade 7 *(n* = 288) and Grade 8 (*n* = 1011) using MI Write in three school districts in the Mid-Atlantic and Southern regions of the United States as part of a randomized controlled trial of MI Write during the 2021–2022 school year. Among the sample, 50.7% (*n* = 658) of students were females, 8.9% (*n* = 116) received special education services (SPED) via an Individualized Education Plan, and 63.4% (*n* = 824) received free-or-reduced price lunch (FRL). Only 3.8% of students qualified for limited English proficiency (LEP). Students were racially and ethnically diverse, including Hispanic (43%), White (25.3%), Black (18.2%), Asian (9.4%), Multiracial (3.3%), American Indian (0.5%), and Pacific Islander (0.4%).

District A, located in a rural setting, has five middle schools, and enrolls approximately 10,000 students. District B, located in a suburban setting, has two middle schools, and enrolls approximately 10,500 students. District C, located in an urban setting, has 21 middle schools, and enrolls nearly 27,000 students. The percentage of students who attain the state-established criterion for reading proficiency in Districts A, B, and C is 26%, 48%, and 50%, respectively.

District B used a district-created writing curriculum that focused on teaching one genre per marking period (i.e., quarterly), beginning with narrative, then literary analysis, then an expository research task, and concluding with a literary analysis. At the time of the study, Districts A and C had both newly adopted the *StudySync* curriculum, which is a web-based ELA curriculum published by McGraw Hill. The curriculum includes six units that address a variety of written genres, including argumentative, informative, explanatory, literacy analysis, and narrative.

It is important to note that the 2021–2022 school year saw the continuation of the COVID-19 pandemic. Educators faced additional challenges during this time, including a combination of in-person and remote instruction, extensive teacher and student absences, the need to make-up missed district and state testing from Spring 2021, teacher burnout, and concerns among teachers of students’ learning loss.

### 2.2 MI Write

MI Write (www.miwrite.com) is developed and marketed by Measurement Incorporated. By providing students with automated feedback and scores, MI Write facilitates the teaching and learning of writing in the classroom (Wilson & Roscoe, 2020). Specifically, MI Write provides an overall (i.e., holistic) score and scores for each of six dimensions of writing quality based on the Six Trait Model (Culham, 2003): idea development, organization, style, sentence fluency, word choice, and conventions. These six traits are grounded in research examining (1) components of effective writing, (2) diagnostic writing assessment, and (3) writing process instruction (for a review see Kozlow & Bellamy, 2004). MI Write further provides automated feedback for each dimension. MI Write has a peer review function and additional skill-building opportunities via interactive multimedia lessons. With MI Write, teachers can customize prompts, message students, and provide in-line and summary comments to supplement AWE feedback. MI Write is thus similar to other contemporary AWE systems that share similar features (see Deeva et al., 2021; Shermis et al., 2016).

### 2.3 Participant use of MI Write

As part of the randomized controlled trial, we asked teachers to assign students monthly MI Write writing activities in which students were expected to complete graphic organizers, draft, and revise their writing, and complete MI Write's interactive lessons; in the latter half of the school year, we asked teachers to additionally require students to peer review each other's writing within MI Write. In sum, across the eight months of study implementation, we intended students to complete eight pre-writing activities (i.e., graphic organizers) and eight essays, revise all eight essays at least twice, complete eight MI Write interactive lessons, and complete three peer reviews. In the end, we provided considerable flexibility around these expectations due to the ongoing COVID pandemic. Teachers primarily utilized MI Write to assign writing prompts with associated graphic organizers and interactive lessons, but teachers rarely assigned peer review within MI Write.

Table 1 shows the extent to which students used MI Write during the school year. In aggregate, students utilized MI Write with low to moderate frequency, primarily to plan and draft their writing and also to edit and revise. Students utilized some of MI Write's supplemental functions, namely peer review and skill-building lessons, but not to a great extent.

**Table 1:** Descriptive statistics of student use of MI Write.

Usage Variables	*M*	Median	Mode	*SD*	Minimum	Maximum	Valid *n*	Missing *n*
Number of prompts completed	3.57	3.00	2	2.34	0	10	1160	139
Number of organizers completed	3.23	3.00	4	2.41	0	10	1160	139
Average number of drafts per prompt	3.23	2.33	1	2.73	1	19.67	1061	238^a^
Prompts completed with at least 2 revisions	1.89	1.00	0	2.38	0	9	1160	139
Number of lessons completed	2.30	1.00	0	3.06	0	11	1160	139
Number of essays reviewed	1.39	0.00	0	2.58	0	15	1160	139

*Note*. ^a^Includes 139 students who had missing data in total and additional 99 students who had zero completed prompts but had other usage as shown in table. A prompt refers to a unique writing assignment created by the teacher. An essay is an individual student's response to that prompt.

On average, students responded to between three and four writing prompts (i.e., separate writing assignments) across the year, and completed approximately three graphic organizers, indicating that students tended to use graphic organizers for each of the assignments they completed. On average, students completed approximately three drafts per prompt—where each draft is defined as an independently scored draft of an essay written in response to a prompt—but the median value was lower (approximately two drafts/essay) as was the mode (one draft/essay), indicating that students solicited MI Write's feedback a low to moderate extent on average.

Furthermore, when students did solicit MI Write's feedback, they more often made edits, (i.e., surface-level changes to a text related to spelling, grammar, or conventions) rather than revisions (i.e., substantive changes to a text; see MacArthur et al., 1991). Using a natural language processing (NLP), we utilized latent semantic analysis to identify a ‘revision’ by comparing a set of two drafts with bag-of-words (BOW) vectorization and cosine distance. Included in BOW were bigram and trigrams as dictionary entries. The closer the resulting cosine distance was to zero, the more similar the drafts. The more recent draft was considered a revision if the cosine distance to the previous revision was at least 0.01. Based on this liberal criterion, the average number of prompts completed with at least two revisions was approximately two with a mode of zero. Thus, students tended to make more edits than substantive revisions when responding to MI Write's automated feedback.

Finally, there was variation in the number of MI Write's skill-building lessons the students completed (range = 0–11). On average, students completed two lessons, but the mode was 0. Usage of MI Write's peer review function was low, which was consistent with teachers' own usage of that function. On average, the students reviewed one essay written by their peers. While several students reviewed up to 15 essays of their peers, the mode was zero.

There were statistically significant differences in students’ use of MI Write across the three districts, as shown in Table 2. Students in District A utilized every feature of MI Write to a greater extent than other districts, whereas the frequency of utilization in District B and District C was not statistically significantly different except for number of organizers completed, average drafts per essay, and number of peer reviews completed. See Table S1 in the supplemental materials for details of these pairwise comparisons.

**Table 2:** Descriptive statistics of student usage by district.

Usage Variables	District A	District B	District C	ANOVA	Pairwise Comparisons
Number of prompts completed	6.02 (2.54)	2.86 (1.63)	3.06 (2.00)	*F*_(2, 1157)_ = 213.83***	A > B=C
Number of organizers completed	5.93 (1.67)	2.25 (1.49)	2.84 (2.43)	*F*_(2, 1157)_ = 268.26***	A > C > B
Average number of drafts per prompt	6.40 (4.06)	2.62 (1.53)	2.21 (1.12)	*F*_(2, 1157)_ = 291.77***	A > B > C
Prompts completed with at least 2 revisions	4.62 (3.08)	1.33 (1.62)	1.15 (1.53)	*F*_(2, 1157)_ = 275.61***	A > B=C
Number of lessons completed	7.26 (3.07)	1.24 (1.12)	0.98 (1.56)	*F*_(2, 1157)_ = 1028.42***	A > B=C
Number of essays reviewed	2.51 (2.05)	0.37 (0.72)	1.73 (3.34)	*F*_(2, 1157)_ = 64.54***	A > C > B

*Note*. Pairwise comparisons conducted with a Bonferroni correction to alpha.

### 2.4 Measures

#### 2.4.1 Dependent measures

We evaluated students' perceptions of MI Write's usability, usefulness, and desirability via a researcher-created online survey administered in Spring 2022. The usability scale included six items (e.g., *In MI Write, it is easy to find the correct writing assignment*), the usefulness scale included six items (e.g., *MI Write helped me plan my writing*), and the desirability scale included two items (*I would recommend MI Write to other students* and *I would like to continue using MI Write*). Table 3 presents the full set of items. Students rated their extent of agreement with each item using a scale of 0 (strongly disagree) to 3 (strongly agree). We based these items on similar items used in past research (Wilson, Huang et al., 2021) and in pilot research conducted immediately prior to the current study. We calculated mean usability, usefulness, and desirability ratings by taking the average of students' ratings across all items for the respective scales. All scales demonstrated high reliability (Cronbach's *α* of 0.74, 0.85, and 0.82, for usability, usefulness, and desirability, respectively).

**Table 3:** Student perceptions of usability, usefulness, and desirability of MI Write.

Item	Overall				District A	District B	District C
	*M* (*SD*)	Median	Mode	*n*	*M* (*SD*)	*M* (*SD*)	*M* (*SD*)
Usability – *In MI Write, it is easy to* …	1.88 (0.49)	2.00	2	1182	2.02 (0.45)	1.79 (0.47)	1.91 (0.51)
1. Find the correct writing assignment.	1.90 (0.65)	2.00	2	1153	2.00 (0.59)	1.77 (0.64)	1.97 (0.66)
2. Locate peer review tasks.	1.85 (0.69)	2.00	2	1038	2.07 (0.65)	1.68 (0.65)	1.89 (0.70)
3. Understand MI Write's feedback.	1.78 (0.73)	2.00	2	1123	1.80 (0.78)	1.73 (0.69)	1.82 (0.75)
4. Use MI Write's graphic organizers.	1.90 (0.74)	2.00	2	1074	2.15 (0.59)	1.81 (0.75)	1.87 (0.76)
5. Understand if my writing received a good score.	2.00 (0.72)	2.00	2	1168	2.11 (0.68)	1.90 (0.71)	2.4 (0.74)
6. Overall, it was easy to use MI Write.	1.94 (0.70)	2.00	2	1188	2.02 (0.73)	1.81 (0.69)	2.02 (0.68)
Usefulness – *MI Write helped me* …	1.98 (0.53)	2.00	2	1187	2.13 (0.43)	1.88 (0.51)	2.00 (0.56)
1. Plan my writing.	1.93 (0.64)	2.00	2	1187	2.09 (0.56)	1.84 (0.62)	1.93 (0.68)
2. Revise my writing.	2.11 (0.67)	2.00	2	1187	2.21 (0.62)	2.07 (0.65)	2.11 (0.70)
3. Learn more about writing.	1.78 (0.75)	2.00	2	1187	1.96 (0.68)	1.65 (0.73)	1.82 (0.78)
4. Know what parts of my writing I should improve.	2.10 (0.72)	2.00	2	1187	2.26 (0.61)	1.99 (0.70)	2.12 (0.76)
5. Keep track of my progress in writing.	2.03 (0.66)	2.00	2	1187	2.19 (0.61)	1.99 (0.63)	2.00 (0.69)
6. Become a better writer.	1.91 (0.77)	2.00	2	1187	2.08 (0.67)	1.76 (0.76)	1.98 (0.79)
Desirability *–*	1.71 (0.77)	2.00	2	1187	1.73 (0.71)	1.52 (0.78)	1.87 (0.74)
1. I would recommend MI Write to other students.	1.84 (0.81)	2.00	2	1187	1.89 (0.80)	1.70 (0.82)	1.97 (0.78)
2. I would like to continue using MI Write.	1.57 (0.86)	2.00	2	1187	1.58 (0.81)	1.35 (0.87)	1.78 (0.82)

*Note*. Response range = 0–3.0 = Strongly Disagree; 1 = Disagree; 2 = Agree; 3 = Strongly Agree.

In addition, we probed students’ perceptions of MI Write qualitatively with an open-ended question: What would you like the MI Write creators to know about how the program can better support students like you? Students could write as much or as little as they wished in response to this question.

#### 2.4.2 Independent measures

##### 2.4.2.1 District context

We accounted for district variation in MI Write usage via fixed effects dummy variables. District C, the most populous district in our sample, was the reference district.

##### 2.4.2.2 Classroom climate

To account for the classroom context, we measured students' perceptions of classroom climate with the *Co-pilot Elevate* scales of *Teacher Caring* and *Classroom Belonging* (PERTS Technical Supplement to Engagement Project Report Brief, n. d.) at two timepoints during the school year, fall and spring, via an online survey. Each scale consisted of three items that asked students to rate, respectively, how much they believed their English Language/Arts (ELA) teacher cared for and respected them (e.g., *My English/Language Arts teacher treats me with respect*) and how much they agreed or disagreed with statements about belonging in their ELA class (e.g., *I have the opportunity to get to know my classmates better in my English/Language Arts class*). Students rated their extent of agreement with the statements using a scale of 0 (strongly disagree) to 3 (strongly agree). We calculated scale values as the average of student's ratings across the three items for each scale. The *Teacher Caring* scale had high reliability in the fall (*α* = 0.82) and spring (*α* = 0.87). The *Classroom Belonging* scale had moderate to high reliability in the fall (*α* = 0.70) and spring (*α* = 0.74).

##### 2.4.2.3 Student demographics

We created dummy variables for the following student demographic factors: female, SPED status, LEP status, and FRL status, and grade level (0 = Grade 7; 1 = Grade 8). Complete demographic data was provided for every student by the respective districts.

##### 2.4.2.4 Students’ writing-related beliefs and affect

We measured three commonly studied and important constructs pertaining to students’ writing-related beliefs and affect: self-efficacy for writing, liking of writing, and recursive process beliefs (see <u>Camacho et al., 2022). Each of these constructs was measured at fall and spring timepoints via an online survey.

Writing self-efficacy refers to students' confidence and self-perceptions of their abilities as writers. Using the *Self-Efficacy for Writing* scale (Bruning et al., 2013), students responded to 19 items in which they rated their confidence for performing certain writing tasks related to applying the conventions of writing (e.g., *I can spell my words correctly*), generating ideas for their writing (e.g., *I can think of many ideas for my writing*), and demonstrating self-regulation of the writing process (e.g., *I can make a good plan for my writing*). Consistent with Bruning et al. (2013), students rated their level of confidence associated with each task on a 0 (not at all confident) to 100 (extremely confident) continuous scale. We calculated a student's average self-efficacy score by taking the average of the student's ratings across the 19 items. The *Self-Efficacy for Writing* scale had high reliability in the fall (*α* = 0.96) and spring (*α* = 0.95).

We administered the *Recursive Process Beliefs* scale, a subscale within the *Beliefs About Writing Survey* developed by Sanders-Reio et al. (2014). This scale includes five items that probe students’ beliefs about the centrality of revising to the writing process (e.g., *Writing requires going back over it to improve what has been written*). Students rated their extent of agreement with each statement on a scale of 0 (strongly disagree) to 4 (strongly agree). The Recursive Process Beliefs scale had moderate reliability in the fall (*α* = 0.66) and spring (*α* = 0.73).

Lastly, we measured students' affect (i.e., feelings) towards writing via the *Liking Writing* scale (Bruning et al., 2013), which consists of four items probing students' enjoyment of writing (e.g., *I enjoy writing*). Students rated their extent of agreement with each statement on a 0 (strongly disagree) to 3 (strongly agree) scale. We averaged student's ratings across the four items to create a scale score, which had high reliability in the fall (*α* = 0.84) and spring (*α* = 0.86).

##### 2.4.2.5 Writing proficiency

Students wrote an essay in response to an argumentative writing prompt in the fall and spring concurrently with the online survey. At each timepoint, this argumentative writing task required students to read two source texts and plan, draft, review, and edit an essay response. Students typed their essays and submitted them online via a survey form. Prompt topics focused on technology in society, with a prompt about “computer-guided robots” administered in the fall and a prompt about “voice-activated assistants” administered in the spring.

We scored students' essays for writing quality using MI Write's automated scoring engine, *Project Essay Grade* (PEG) (Wilson, Huang et al., 2021; Page, 2003). PEG utilizes grade-band specific (Grade 3–4, 5–6, 7–8, 9–10, 11–12) and genre-specific (narrative, informative, argumentative) scoring algorithms to evaluate responses to both system and customized prompts. PEG's automated six-trait scoring model has very high internal consistency (*α*_fall_ = 0.995; *α*_spring_ = .996). Hence, for the purposes of this study we measured students' writing proficiency using the Overall Score provided by PEG, which is the sum of the six trait scores and ranges from 6.0 to 30.0 points. The Overall Score is highly reliable (see Chen, Hebert, & Wilson, 2022, Wilson, Chen, Sandbank, & Hebert, 2019).

### 2.5 Data analysis

To answer RQ1, we calculated descriptive statistics for survey items and scales probing students' perceptions of MI Write's usability, usefulness, and desirability. We completed a thematic analysis of the open-ended survey responses. We applied a deductive approach, using a priori coding based on our social validity framework of usability, usefulness, and desirability.

To answer RQ2, we utilized hierarchical (i.e., block) entry regression models to examine which factors uniquely predicted students' ratings of MI Write's usability, usefulness, and desirability. We input the predictors in five blocks using SPSS V.28: Block 1–district context; Block 2–classroom climate; Block 3–student demographics; Block 4–writing-related beliefs and affect; and Block 5–writing proficiency. For each outcome variable, we estimated two regression equations: one using independent variables measured in the fall prior to treatment, and another using independent variables measured in the spring following treatment. In this way, we could identify whether a stable subset of variables consistently predicted students' perceptions. In total, we estimated six regression models. We report standardized regression coefficients (β) as a measure of effect size. According to Cohen's (1988) interpretation of standardized coefficients, we identify 0.05 as a small effect, 0.10 as a medium effect, and 0.25 as a large effect.

#### 2.5.1 Handling of missing data

The rate of missingness across fall predictors ranged from 5% (e.g., self-efficacy) to 13% (i.e., writing proficiency); the rate of missingness across spring predictors ranged from 8% (e.g., self-efficacy) to 17% (i.e., writing proficiency). The rate of missingness for the three dependent variables (usability, usefulness, and desirability) remained at 9%. As is appropriate when estimating regression models (see Hughes et al., 2019), we elected to use listwise deletion to handle missing data under the assumption that missingness was independent of the outcome variable, after accounting for the predictor variables. Nevertheless, we conducted a sensitivity analysis using mean imputation and results were commensurate.

## 3 Results

### 3.1 Research question 1: student perceptions of MI write

Table 3 presents descriptive statistics for student perceptions of the usability, usefulness, and desirability of MI Write.

#### 3.1.1 Usability

Overall, students rated MI Write as moderately easy to use (*M* = 1.88; *SD* = 0.49; mode = 2). Indeed, in response to the open-ended survey item, many students commented on MI Write's usability positively, such as: “I like how MI Write is easy to use” and “I think MI Write is great and easy to use.” However, there were significant differences in usability ratings by district [*F*_(2, 1179)_ = 19.32, *p* < .001]. Pairwise comparisons using a Bonferroni adjusted alpha level of 0.016 (0.5/3) indicated that students in District A expressed significantly higher ratings than students in District B and District C, and students in District C expressed significantly higher ratings than students in District B (A > C > B). See Table S2 in the supplemental materials for full information.

As shown in Tables 3 and in terms of specific aspects of MI Write's usability, students most strongly agreed that it was easy to understand if their writing received a good score. Several students elaborated on this point. For example, “MI Write helps students like me who do not know how to make their writing better because when you submit your writing, it gives you a score in each category.” Students least strongly agreed that it was easy to understand MI Write's feedback. For example, students expressed: “I don't really understand the feedback that I get, so sometimes I don't know what to change” and “I would like the MI write creators to know that the feedback given automatically is very vague and limited.” Thus, generally, students found MI Write easy to use, but found its scoring easier to understand than its feedback.

#### 3.1.2 Usefulness

Overall, students agreed that MI Write was useful (*M* = 1.98; *SD* = 0.53; mode = 2) and reported positive comments, such as: “I believe that MI Write is a good program to help students my age improve their writing and also a program that takes us, the students, through the writing process, which guides us to becoming better writers,” and “It helps everyone with planning and revising essays.” However, there were significant differences in usefulness ratings by district [*F*_(2, 1184)_ = 17.92, *p* < .001]. Pairwise comparisons using Bonferroni correction indicated that students in District A expressed significantly higher ratings than students in District B and District C, and students in District C expressed significantly higher ratings than students in District B (A > C > B). See Table S2 in the supplemental materials.

Specifically, as shown in Table 3, students most strongly agreed that MI Write helped them revise their writing and know what parts of their writing to improve. For example, students wrote that “[MI Write] helps us see where we made a mistake and lets us go back and fix it,” and “It made me realize what I should work on, such as grammar, idea placement, and how good my current writing is. It made me better in my writing.” Students least strongly agreed that MI Write could help them learn more about writing. Comments included: “Rather than saying what is wrong with the writing, [MI Write should] teach it and explain it,” and MI Write would be improved if it had a “Tips and Tricks on how to get better at writing and how to show us to use [things] in a correct way.” Thus, students generally found MI Write to be useful, but more so for identifying areas of improvement than learning how to address them.

#### 3.1.3 Desirability

Overall, students rated MI Write's desirability lower than its usability and usefulness (*M* = 1.71; *SD* = 0.77; mode = 2). There were significant differences across districts with respect to desirability [*F*_(2, 1184)_ = 26.76, *p* < .001]. Pairwise comparisons using a Bonferroni correction indicated that students in District A expressed significantly higher desirability ratings than students in District B but not those in District C, and students in District C expressed significantly higher ratings than students in District B (A = C > B). Specifically, as shown in Table 3, students more strongly agreed that they would recommend MI Write to other students than they would like to continue using MI Write. See Table S2 in the supplemental materials.

Students often wrote positive comments about MI Write, such as: “I think the program is a great experience, especially for people who are brand new to it” and “I really like MI Write and it's a great program.” Frequently, students positively commented on MI Write's desirability relative to its perceived usefulness. For instance: “I like MI Write and it helps me understand my essays and correct me when I am wrong.”

Similarly, when students criticized MI Write's desirability, they often referenced utility/usefulness. For example, one student wrote: “Personally, I feel that MI Write is another complicated teaching tool. … From the student's perspective, it doesn't really provide any unique tools that cannot be figured out on a pen and paper.” Also, negative comments about desirability were associated with negative perceptions about the accuracy of the automated scoring and feedback. For instance, students shared comments like: “Sometimes the corrections being given are invalid. The system doesn't pick up on certain things being said and why. The use of the word may be correct when the system marks it wrong,” and “Words that are spelled correctly get marked wrong and it's pretty frustrating when it comes to getting a bad score because of that. MI Write is an okay space for writing, but honestly, I'm not a big fan of it.” Thus, generally, students tended to agree that MI Write was a desirable tool, but their perceptions about its usefulness and of its scoring and feedback accuracy influenced their perceptions.

### 3.2 Research question 2: predictors of students’ perceptions

#### 3.2.1 Descriptive statistics

Table 4 presents descriptive statistics for the independent variables. Students tended to agree that their ELA teacher was caring, and they felt a sense of belonging in their ELA classroom. Students were somewhat confident about their own writing ability (*M*_fall_ = 64.76; *M*_spring_ = 69.64). They tended to dislike writing (*M*_fall_ = 1.73; *M*_spring_ = 1.67) but held strong beliefs about writing as a recursive process (*M*_fall_ = 3.00; *M*_spring_ = 3.11). Finally, students’ argumentative writing quality, as measured by the PEG Overall Score, showed that the students had lower proficiency in the fall (*M* = 15.93; *SD* = 4.70) than in the spring (*M* = 17.28; *SD* = 4.91).

**Table 4:** Descriptive statistics of independent variables measured at fall and spring.

Variables	*M*	*SD*	Minimum	Maximum
Fall Predictors				
Teacher caring	2.34	0.60	.00	3.00
Classroom belonging	2.03	0.61	.00	3.00
Self-efficacy	64.76	21.10	.89	100.00
Liking writing	1.73	0.68	.00	3.00
Recursive process beliefs	3.00	0.51	.60	4.00
Writing proficiency	15.93	4.70	6.00	27.81
Spring Predictors				
Teacher caring	2.29	0.71	.00	3.00
Classroom belonging	2.07	0.65	.00	3.00
Self-efficacy	69.64	19.31	7.68	100.00
Liking writing	1.67	0.68	.00	3.00
Recursive process beliefs	3.11	0.54	.00	4.00
Writing proficiency	17.28	4.91	6.00	27.30

*Note.* Fall and spring Teacher Caring and Classroom Belonging scales: range = 0 (strongly disagree) – 3 (strongly agree). Fall and spring Self Efficacy scale: range = 0 (not at all confident) – 100 (extremely confident). Fall and spring Liking Writing scales: range = 0 (strongly disagree) – 3 (strongly agree). Fall and spring Recursive Process Beliefs scales: range = 0 (strongly disagree) – 4 (strongly agree). Fall and spring writing proficiency measured via PEG Overall Score (range = 6–30) applied to argumentative essays.

#### 3.2.2 Correlations

Table 5 presents correlations among dependent and independent variables at fall (below diagonal) and spring (above diagonal). Usability and usefulness were moderately correlated (*r* = 0.62), as were usefulness and desirability (*r* = 0.64), but less so usability and desirability (*r* = 0.50), indicating that students’ perceptions were differentiated across these constructs.

**Table 5:** Correlation matrix of dependent and independent variables.

Measure	1	2	3	4	5	6	7	8	9	10	11	12	13	14	15	16
1. Usability	–	.617**	.496**	−.158**	.136**	.185**	.167**	.045	−.019	.051	.074*	.108**	.270**	.307**	.281**	.041
2. Usefulness	.617**	–	.635**	−.144**	.142**	.180**	.206**	.028	−.014	.061*	.041	.028	.210**	.338**	.377**	.002
3. Desirability	.496**	.635**	–	−.197**	.014	.154**	.170**	.048	−.018	.071*	.102**	.015	.148**	.353**	.280**	.035
4. District A	−.158**	−.144**	−.197**	–	−.374**	.123**	.123**	−.056*	−.031	−.156**	−.262**	−.108**	−.064*	−.172**	−.047	.013
5. District B	.136**	.142**	.014	−.374**	–	−.121**	−.153**	.047	.024	.037	−.124**	.256**	−.083**	−.012	.002	−.025
6. Teacher caring	.150**	.148**	.102**	.067*	−.176**	–	.701**	−.005	−.046	−.031	−.054	−.110**	.174**	.153**	.186**	.077*
7. Classroom belonging	.119**	.139**	.086**	.048	−.189**	.672**	–	.013	−.054	−.037	−.071*	−.120**	.236**	.206**	.178**	.064*
8. Female	.045	.028	.048	−.056**	.047	.067*	.011	–	−.078**	−.035	.018	.029	−.008	.194**	.066*	.142**
9. SPED	−.019	−.014	−.018	−.031	.024	−.024	−.031	−.078**	–	−.020	.107**	−.010	−.186**	−.029	−.107**	−.252**
10. LEP	.051	.061*	.071*	−.156**	.037	−.054	−.025	−.035	−.020	–	.085**	−.076**	−.079**	.025	−.018	−.062*
11. FRL	.074*	.041	.102**	−.262**	−.124**	−.069*	−.051	.018	.107**	.085**	–	−.090**	−.059*	.006	−.066*	−.104**
12. Grade	.108**	.028	.015	−.108**	.256**	−.066*	−.087**	.029	−.010	−.076**	−.090**	–	.059*	−.005	.048	.203**
13. Self-efficacy	.144**	.110**	.082**	−.034	−.168**	.250**	.322**	.006	−.194**	−.029	−.033	.038	–	.463**	.236**	.255**
14. Liking writing	.146**	.180**	.205**	−.110**	−.132**	.233**	.284**	.185**	−.023	−.008	.058*	−.040	.487**	–	.255**	.206**
15. Recursive process beliefs	.149**	.176**	.161**	−.059*	−.128**	.232**	.229**	.043	−.110**	−.024	−.032	.066*	.332**	.275**	–	.120**
16. Writing proficiency	.010	−.034	.010	−.002	−.099**	.152**	.177**	.148**	−.227**	−.052	−.159**	.194**	.314**	.213**	.175**	–

*Note.* Correlation of fall predictors with outcome variables in lower diagonal. Correlation of spring predictors with outcome variables in upper diagonal. SPED = Students who receive special education services with an individualized education plan (IEP). LEP = Students with limited English proficiency. FRL = students eligible for free-and-reduced lunch.

**p < .01.

Fall variables tended to have weak correlations with outcomes, ranging from 0.01 to 0.15. Spring predictors had stronger correlations with outcomes, but were still weak overall, ranging from 0.00 to 0.35. Among the independent variables at both fall and spring, attitudinal measures were significantly correlated with each other and with writing proficiency.

#### 3.2.3 Regression models predicting usability

Regression models including fall and spring predictors explained 12.1% and 23% of the variance in students’ MI Write usability ratings, respectively. VIF values (1.002–1.269) for these models indicated absence of multicollinearity.

The first block of predictors (district context) explained 4.5% of the variance, *F*_Δ_(2, 1045) = 24.40, *p* < .001, in the model with fall predictors and 4.3% of the variance, *F*_Δ_(2, 1061) = 23.73, *p* < .001, in the model with spring predictors.

The second block (classroom climate) explained an additional 3.6% of the variance, *F*_Δ_(2, 1043) = 20.48, *p* < .001, in the model with fall predictors and an additional 4.5% of the variance, *F*_Δ_(2, 1059) = 26.33, *p* < .001, in the model with spring predictors.

The third block (student demographics) explained an additional 2.0% of the variance, *F*_Δ_(5, 1038) = 4.69, *p* < .001, in the model with fall predictors and an additional 2.1% of the variance, *F*_Δ_(5, 1054) = 4.98, *p* < .001, in the model with spring predictors.

The fourth block (writing-related beliefs and affect) explained an additional 1.6% of the variance, *F*_Δ_ (3, 1035) = 6.30, *p* < .001, in the model with fall predictors and an additional 11.7% of the variance, *F*_Δ_(3, 1051) = 52.73, *p* < .001, in the model with spring predictors.

The fifth block (writing proficiency) explained an additional 0.4% of the variance, *F*_Δ_(1, 1034) = 4.37, *p* = .037, in the model with fall predictors and an additional 0.4% of the variance, *F*_Δ_(1, 1050) = 5.22, *p* = .022 in the model with spring predictors.

The results of the hierarchical regression models indicate that the strongest blocks of fall predictors were district effects followed by classroom climate, whereas the strongest blocks of spring predictors were writing-related beliefs and affect and classroom climate. In both the fall and spring, writing proficiency explained negligible variance in student perceptions of usability despite adding uniquely to the overall regression model.

As shown in Table 6 and Table S3 in the supplemental materials, the following variables were significant predictors of usability ratings both prior to students' exposure to MI Write (fall) and following treatment (spring): district fixed effects, teacher caring, LEP status, FRL status, grade level, liking writing beliefs, recursive process beliefs, and writing proficiency. However, writing proficiency was negatively related to student perceptions of usability: students with higher MI Write scores tended to have lower ratings of MI Write's usability. Classroom belonging, gender, and SPED status were consistently unrelated to usability ratings.

**Table 6:** Standardized regression coefficients for final linear regression model for all outcomes.

	Usability (Fall predictors)	Usability (Spring predictors)	Usefulness (Fall predictors)	Usefulness (Spring predictors)	Desirability (Fall predictors)	Desirability (Spring predictors)
District A	.149***	.159***	.191***	.180***	−.004	.000
District B	−.085*	−.036	−.048	−.007	−.169***	−.135***
Teacher caring	.137***	.109**	.109**	.057	.086*	.069
Classroom belonging	.039	.030	.056	.100**	−.016	.069
Female	.014	.001	−.022	−.036	.003	−.038
SPED	−.018	.007	−.025	.000	−.037	−.031
LEP	.078**	.061*	.050	.041	.049	.048
FRL	.085**	.115***	.060	.094***	.073	.094**
Grade	.100***	.107***	.034	.022	.047	.055
Self-efficacy	.050	.160***	−.019	.045	−.054	−.063
Liking writing	.071*	.156***	.140***	.216***	.161***	.293***
Recursive process beliefs	.086**	.211***	.143***	.299***	.119***	.200***
Writing proficiency	−.069*	−.068*	−.096**	−.084**	−.053	−.043

*Note*. *n* = 1048 for fall and *n* = 1064 for spring models. Writing proficiency measured using the MI Write Overall Score (range = 6–30).

*p ≤ .05.

**p ≤ .01.

***p ≤ .001.

There were some differences between the fall and spring prediction models. Self-efficacy for writing significantly predicted usability in the spring but not in the fall. The strength of some predictors, indicated by their standardized coefficient (β), varied from fall to spring. Specifically, teacher caring and LEP status were less important predictors following treatment in the spring, while FRL status, liking writing, and recursive process beliefs exhibited relative increases in importance in the spring.

#### 3.2.4 Regression models predicting usefulness

The regression models including fall and spring predictors explained 12.1% and 25.4% of the variance in students’ MI Write usefulness ratings, respectively. VIF values (1.003–1.272) for these models indicated absence of multicollinearity.

The first block of predictors (district context) explained 3.7% of the variance, *F*_Δ_(2, 1049) = 20.34, *p* < .001, in the model with fall predictors and 3.3% of the variance, *F*_Δ_(2, 1063) = 18.27, *p* < .001, in the model with spring predictors.

The second block (classroom climate) explained an additional 3.5% of the variance, *F*_Δ_(2, 1047) = 19.96, *p* < .001, in the model with fall predictors and an additional 5.8% of the variance, *F*_Δ_(2, 1061) = 33.95, *p* < .001, in the model with spring predictors.

The third block (student demographics) explained an additional 0.7% of the variance, *F*_Δ_(5, 1042) = 1.56, *p* = .168, in the model with fall predictors and an additional 0.6% of the variance, *F*_Δ_(5, 1056) = 1.46, *p* = .200, in the model with spring predictors. The inclusion of student demographic variables did not uniquely add to the strength of either regression model.

The fourth block (writing-related beliefs and affect) explained an additional 3.4% of the variance, *F*_Δ_(3, 1039) = 13.43, *p* < .001, in the model with fall predictors and an additional 15.1% of the variance, *F*_Δ_(3, 1053) = 70.50, *p* < .001, in the model with spring predictors.

The fifth block (writing proficiency) explained an additional 0.7% of the variance, *F*_Δ_(1, 1038) = 8.57, *p* = .003 in the model with fall predictors and an additional 0.6% of the variance, *F*_Δ_(1, 1052) = 8.18, *p* = .004, in the model with spring predictors.

Results indicate that the strongest blocks of fall predictors were district effects followed by classroom climate, whereas the strongest blocks of spring predictors were students’ writing-related beliefs and affect followed by classroom climate. In both the fall and spring, student demographic factors did not contribute to the strength of the model, whereas writing proficiency explained negligible variance in student perceptions of usefulness but did add uniquely to the overall regression models.

As shown in Table 6 and Table S4 in the supplemental materials, the following variables were significant predictors of usefulness ratings both prior to students’ exposure to MI Write (fall) and following treatment (spring): district fixed effects, liking writing, recursive process beliefs, and writing proficiency. However, writing proficiency was negatively related to student perceptions of usefulness, as was the case when predicting usability perceptions. None of the student demographics variables significantly predicted usefulness ratings, except for FRL status, which was a significant predictor in the spring model only. Also, self-efficacy for writing was consistently unrelated to usefulness ratings.

There were some differences between the fall and spring prediction models. One measure of classroom climate in the fall—teacher caring—predicted usefulness ratings, whereas in the spring, the other measure of classroom climate, classroom belonging, predicted usefulness ratings. The strength of some predictors, indicated by their standardized coefficient (β), varied from fall to spring. Liking writing and recursive process beliefs increased in relative importance, with the latter exhibiting a doubling of effect size from fall to spring.

#### 3.2.5 Regression models predicting desirability

The regression models with fall and spring predictors explained 10.4% and 21.2% of the variance in students’ MI Write desirability ratings, respectively. VIF values (1.002–1.272) for these models indicated absence of multicollinearity.

The first block of predictors (district context) explained 4.8% of the variance, *F*_Δ_(2, 1049) = 26.73, *p* < .001, in the model with fall predictors and 4.8% variance, *F*_Δ_(2, 1063) = 26.74, *p* < .001, in the model with spring predictors.

The second block (classroom climate) explained an additional 1.1% of the variance, *F*_Δ_(2, 1047) = 6.20, *p* = .002, in the model with fall predictors and an additional 3.7% of the variance, *F*_Δ_(2, 1061) = 21.60, *p* < .001, in the model with spring predictors.

The third block (student demographics) explained an additional 1.0% of the variance, *F*_Δ_(5, 1042) = 2.31, *p* = .043, in the model with fall predictors and an additional 1.1% of the variance, *F*_Δ_(5, 1056) = 2.46, *p* = .031, in the model with spring predictors.

The fourth block (writing-related beliefs and affect) explained an additional 3.2% of the variance, *F*_Δ_ (3, 1039) = 12.49, *p* < .001, in the model with fall predictors and an additional 11.5% of the variance, *F*_Δ_(3, 1053) = 51.24, *p* < .001, in the model with spring predictors.

The fifth block (writing proficiency) explained an additional 0.2% of the variance, *F*_Δ_(1, 1038) = 2.52, *p* = .113, in the model with fall predictors and an additional 0.2% of the variance, *F*_Δ_ (1, 1052) = 2.01, *p* = .156, in the model with spring predictors. In both models, writing proficiency did not statistically significantly improve model prediction, explaining less than 1% of the variance in students’ desirability ratings.

Results indicate that the strongest set of predictors in the fall model were district fixed effects followed by students’ writing-related beliefs; the inverse was true for the spring model.

As shown in Table 6 and Table S5 in the supplemental materials, the following variables were significant predictors of desirability ratings both prior to students' exposure to MI Write (fall) and following treatment (spring): district fixed effects, students' liking writing beliefs, and recursive process beliefs. Classroom climate showed little relations with students’ desirability ratings: classroom belonging was not a significant predictor at either timepoint and teacher caring was a significant predictor in the fall only. In addition, the following student demographics showed no relations with desirability ratings: gender, SPED status, LEP status, and grade level. FRL status was a significant predictor in the spring only. Self-efficacy for writing and writing proficiency were also consistently unrelated to desirability ratings. Again, the strength of some predictors, indicated by their standardized coefficient (β), varied from fall to spring. Liking writing and recursive process beliefs increased in relative importance, with the effect sizes of both variables nearly doubling from fall to spring.

## 4 Discussion

Little research has examined students' perceptions of AWE, particularly the perceptions of L1 and K–12 students (Fu et al., 2022). Moreover, no prior research has attempted to systematically and quantitatively identify multiple factors that predict students' perceptions of AWE. Identifying such factors may assist in identifying populations of students who are prone to perceive AWE as beneficial and populations who may require additional support to perceive benefits. Thus, in the present study, after summarizing students' perceptions of the MI Write AWE system, we identified factors that predicted students’ perceptions of AWE and determined the extent to which those factors varied following a year of AWE implementation.

Importantly, while the study occurred within the context of ongoing effects of the COVID-19 pandemic, results pointed to factors at the system-level (AWE), district-level, classroom-level, and individual-level that were not specific to that context, but more universally applicable to diverse educational settings. These factors, including the perceived usability and usefulness of the MI Write AWE system, perceptions of teacher caring, FRL status, writing-related beliefs, and writing performance, transcend the challenges posed by the pandemic. This broader scope of applicability suggests that the findings and implications of this research are relevant and valuable when considering AWE implementation generally.

### 4.1 Students’ perceptions

Students' perceptions of MI Write tended to be positive. On average, students agreed (though not strongly) that MI Write was useable, useful, and desirable. Nevertheless, consistent with prior research (e.g., Wilson, Huang et al., 2021), students were able to differentiate between distinct aspects of AWE's overall social validity, evidenced by distinct usability, usefulness, and desirability ratings. Indeed, correlations among these scales at most were moderate. Usability was more strongly correlated with usefulness than with desirability; however, desirability was more strongly related to perceptions of usefulness.

Findings indicate that the perceived usability of AWE may be less relevant to overall social validity perceptions than perceived utility. Indeed, students' open-ended survey responses appeared to confirm the strength of these associations. Often, when students gave a reason for their positive or negative appraisal of MI Write's desirability, they also commented on its usefulness in a parallel manner. Students who appraised MI Write's scoring and feedback as accurate and useful tended to remark positively about MI Write's usefulness and desirability; the inverse was also observed. With respect to specific aspects of MI Write perceived as most useful, most students agreed that MI Write was beneficial for revising and identifying areas of improvement in their writing but expressed a desire for more instructional guidance and tips on how to address those areas. Findings are consistent with prior research documenting a relationship between appraisals of AWE scoring accuracy and feedback utility and perceptions of AWE systems in general (Fu et al., 2022; Roscoe et al., 2018, Roscoe, Wilson, Johnson, & Mayra, 2017).

### 4.2 Factors predicting students’ perceptions

First, student perceptions of usability, usefulness, and desirability varied across districts, with district fixed effects consistently predicting each of these outcomes. There are several possible explanations for this finding. Differences in perceptions may be due to unexamined differences across district contexts during the study period. Differences in perceptions may also or instead be due to district-specific differences in implementation (see Table 2). Indeed, prior research indicates that AWE implementation varies across districts (Deane et al., 2021; Mayfield & Butler, 2018). Moreover, AWE implementation occurs within a curricular context, and teachers may perceive some curricula as being less amenable to AWE integration (see Link et al., 2014) or teachers may selectively utilize certain AWE functions, such as its writing practice versus peer review functionality, as occurred in the present study and in prior research (Wilson, Huang et al., 2021). Future research should more carefully measure not only overall usage rates across districts, as we did in the present study, but specific implementation methods, such as using AWE to supplement or deliver core instruction (c.f., Wilson et al., 2022). A fruitful area of future AWE research would be developing a questionnaire and observation protocol to classify different implementation approaches and identify which specific AWE features are utilized by teachers in each approach. Once classified, analyses can examine associations between these different implementation approaches and students’ social validity perceptions.

Second, after controlling for other factors, students' perceptions of their classroom climate, particularly of Teacher Caring, positively predicted AWE perceptions. Students' initial (fall) perceptions of Teacher Caring consistently and positively predicted usability, usefulness, and desirability. AWE is part of the instructional context, and prior research suggests that students’ initial impression of their teacher is a reliable and critical indicator of a positive instructional context (Everston & Emmer, 1982; Mainhard et al., 2011), potentially explaining this finding.

Third, after controlling for other factors, certain demographic variables exhibited notable predictive power. LEP status, FRL status, and grade level positively predicted usability both prior to and after using AWE (i.e., in the fall and spring models), and FRL status positively predicted usefulness and desirability, but only in the spring models. In contrast, gender and special education status did not predict students' AWE perceptions at either timepoint. Such results are encouraging with respect to the inclusiveness of MI Write, particularly for its use with student groups that have historically struggled to attain writing proficiency (Deane, 2023). Students with LEP and students qualifying for FRL perceived MI Write to be more useable (fall and spring), and more useful and desirable (spring only). The positive perceptions of MI Write by students with LEP and those qualifying for FRL across both fall and spring models might be rooted in their perceived need for rapid feedback. The AWE system's ability to provide consistent and immediate feedback could be particularly valuable for these groups, enhancing its perceived social validity.

With respect to gender and special education status, the design and functionality of AWE systems, including MI Write, aim for universal accessibility, which might reduce potential disparities in perception based on these demographic factors. For instance, a gender-neutral interface and feedback design could result in uniform perceptions irrespective of gender. Similarly, the consistent support the system offers to students with special education needs might align with the support provided to general education students, leading to minimal perceptual differences. Researchers should continue to examine how and why perceptions, usage, and outcomes of AWE implementation may vary according to student demographics, particularly socioeconomic status.

Fourth, after controlling for other factors, students' writing-related beliefs and affect, particularly Liking Writing and Recursive Process beliefs, positively predicted their perceptions of AWE. Students who liked writing more and more strongly believed in the centrality of revision to the writing process held more positive perceptions of MI Write's usability, usefulness, and desirability both prior to and after using MI Write. Indeed, these variables had the largest effect sizes among the examined predictors. Writing well, particularly revising, requires motivation (Deane, 2018; Graham, 2018). Thus, this finding may likely indicate greater acceptance of AWE within instruction for those who already possess motivation to write.

Surprisingly, despite its connection to writing proficiency (Graham et al., 2018) and motivation (Camacho et al., 2022), self-efficacy did not significantly predict perceptions. This divergence suggests that although self-efficacy impacts writing performance, it might not directly shape perceptions of a tool's usability or desirability. This unexpected result could be partly attributed to the mandatory use of the AWE system in this study, as teachers required their students to use it. In such a setting, students' self-belief in their writing capabilities might not heavily influence their views on the system. If, however, students had the autonomy to decide whether to use AWE, their self-efficacy might play a greater role in that decision. For instance, students with lower writing self-efficacy might lean more towards using the system for feedback, while those with higher self-efficacy might not see the need for it.

Future research should explore relationships between self-efficacy and other writing-related beliefs and affect in the context of voluntary AWE adoption in a naturalistic setting to compare results from the present study, which focused on compulsory AWE implementation in the context of a randomized controlled trial. Such investigations would provide insights into the generalizability of our findings and help discern the nuances of student perceptions in different instructional contexts. In addition, researchers and AWE developers should continue to explore how students with different attitudes and beliefs about writing perceive and utilize AWE, as well as identifying methods of promoting more positive attitudes and adaptive beliefs about writing amongst students who use AWE.

Finally, after controlling for other factors, students' writing proficiency consistently negatively predicted their perceptions of MI Write's usability and usefulness; writing proficiency was not related to perceptions of desirability. Students with greater writing proficiency perceived MI Write to be less easy to use and less useful, consistent with prior research (Huang & Renandya, 2020; Ranalli et al., 2017). Based on students' open-ended survey responses, one possibility might be that more proficient writers did not receive the feedback they needed from MI Write, perhaps due to noted limitations in AWE feedback on higher-level writing skills (Deane, 2013). Researchers and AWE developers should continue to expand the assessment and feedback capabilities of their systems to provide feedback on higher-level writing skills (e.g., Correnti et al., 2022; Raković et al., 2021), as well as aspects of the writer's process, not just the writing product (see Deane et al., 2021; Raković et al., 2022).

#### 4.2.1 Differences between fall and spring models

Findings generally pointed to more pronounced associations between measures in the spring after students had spent time using the AWE system, compared to the fall when they were initially introduced to it. Two key explanations emerge for this observation. Firstly, the timing of the AWE perceptions survey plays a role: measures taken at the same time tend to exhibit stronger correlations than those spaced months apart. Secondly, and closely related, is the possibility that malleable factors, like writing-related beliefs, affect, and proficiency, evolved in response to extended system use and instructional exposure. While we highlighted differences in predictors that showed varied significance between fall and spring, our primary focus was on those factors that consistently predicted perceptions across both time points. Such consistent predictors are likely to represent stable factors that significantly influence students' perceptions of AWE.

### 4.3 Limitations and future directions

Several limitations should be considered when interpreting the results of this study. We examined the perceptions of students in Grades 7 and 8 who used the AWE system MI Write exclusively. MI Write includes representative features of AWE including natural language processing, automated scores and feedback, and recursive writing practice supports, sharing these features with other popular AWE tools such as Criterion, Pigai, Grammarly, My Access, Writing Pal, and e-rater (see Deeva et al., 2021; Shermis et al., 2016). However, more research is needed to examine student perceptions of other AWE systems, particularly among K–12 and L1 students, to understand the generalizability of study findings.

Second, student participants were racially and ethnically diverse, including a relatively high proportion of Black and Hispanic students. Most students were eligible for FRL. We consider this a strength of our study, but results may not generalize to other populations. Third, it is reasonable that the communicative purposes and genres of the tasks that students completed with MI Write may have been related to their perceptions. We did not have access to data to allow us to explore such a relationship, but future research should do so.

Fourth, while our prediction models incorporated a comprehensive set of variables grounded in prior research and theory, a notable portion of variance in student perceptions remains unexplained. This unaccounted variance suggests the potential influence of unmeasured factors, such as the complexity of how students utilize and interact with AWE, personal histories with writing feedback and evaluation, or even transient situational factors. Achieving full explanatory power is challenging due to the complex interplay of cognitive, affective, and situational factors. Thus, although this study has identified salient factors that may influence students' AWE perceptions, future research should explore other predictors that may explain additional variability in students’ perceptions of AWE.

One potential avenue for future research involves the integration of quantitative measures of writing performance with students’ perceptions. By conducting comparative analyses of writing samples before and after engagement with the AWE system, researchers can quantitatively assess changes in writing quality and skill development. This approach would allow for ascertaining whether perceived usefulness of the platform corresponds to tangible enhancements in academic writing proficiency. Furthermore, the incorporation of qualitative research methods can provide a more nuanced exploration of the relationship between perceived usefulness and writing skill development. Focus groups or interviews with students who have interacted with the AWE system could be conducted to gather in-depth insights into their experiences. These qualitative methods would enable researchers to uncover underlying factors that contribute to the perceived impact on writing development and gain a more holistic understanding of the dynamics at play.

## 5 Conclusion

This study is the first to identify predictors of students' perceptions of AWE systematically and quantitatively. Findings indicate that several factors help explain variability in students’ perceptions of AWE—district context, classroom climate (particularly Teacher Caring), FRL status, liking writing, recursive process beliefs, and writing proficiency—and other factors do not, such as gender and special education status. Generally, less proficient writers and those with more motivation towards writing reported more positive perceptions of AWE. Based on this knowledge, researchers, practitioners, and AWE developers might take two steps: (a) develop screening instruments and questionnaires that can be administered in advance of implementing AWE with students, the findings of which may inform the development of differentiated AWE training and instructional supports; and (b) expand AWE functionality so that a greater number of students perceive AWE as a useable, useful, and desirable tool to support learning.

## CRediT authorship contribution statement

**Joshua Wilson:** Conceptualization, Formal analysis, Funding acquisition, Investigation, Methodology, Project administration, Supervision, Writing - original draft, Writing - review & editing. **Fan Zhang:** Formal analysis, Methodology, Writing - original draft, Writing - review & editing. **Corey Palermo:** Conceptualization, Funding acquisition, Methodology, Project administration, Resources, Supervision, Writing - review & editing. **Tania Cruz Cordero:** Investigation, Methodology, Writing - review & editing. **Matthew C. Myers:** Investigation, Methodology, Writing - review & editing. **Halley Eacker:** Investigation, Project administration, Resources, Supervision. **Andrew Potter:** Investigation, Methodology. **Jessica Coles:** Investigation, Resources.

## Declaration of competing interest

The following authors declare no conflicts of interest relevant to this work: Joshua Wilson, Fan Zhang, Tania Cruz Cordero, Matthew C. Myers, Andrew Potter. The following authors are employed by Measurement Incorporated: Corey Palermo, Halley Eacker, Jessica Coles.

## Data Availability

Data will be made available on request.
