# Supplementary material for: Predictors of middle school students’ perceptions of automated writing evaluation
Source: Comput Educ. 2024 Apr;211:104985. doi: 10.1016/j.compedu.2023.104985 (PMC10839244; doi:10.1016/j.compedu.2023.104985)
Supplement: Multimedia component 1 [file mmc1.docx]

**Predictors of Middle School Students’ Perceptions of Automated Writing Evaluation**

***Supplemental Materials***

Table S1

*Pairwise Comparisons of District Differences in Students’ Mean Usability, Usefulness, and Desirability Ratings*

|  | District A–B | | | | District B–C | | | | District A–C | | | |
| --- | --- | --- | --- | --- | --- | --- | --- | --- | --- | --- | --- | --- |
|  | Mean Difference | *SE* | *p* | 95% CI | Mean Difference | *SE* | *p* | 95% CI | Mean Difference | *SE* | *p* | 95% CI |
| Prompts completed | 3.157 | .165 | <.001 | [2.76, 3.55] | -0.197 | .132 | .400 | [-0.51, 0.12] | 2.960 | .159 | <.001 | [2.58, 3.34] |
|  |  |  |  |  |  |  |  |  |  |  |  |  |
| Organizers completed | 3.675 | .164 | <.001 | [3.28, 4.07] | -0.583 | .131 | <.001 | [-0.90, -0.27] | 3.092 | .159 | <.001 | [2.71, 3.47] |
|  |  |  |  |  |  |  |  |  |  |  |  |  |
| Average drafts/essay | 3.772 | .184 | <.001 | [3.33, 4.21] | 0.410 | .152 | .021 | [0.045, 0.77] | 4.181 | .181 | <.001 | [3.75, 4.62] |
|  |  |  |  |  |  |  |  |  |  |  |  |  |
| Prompts with 2 revisions | 3.294 | .162 | <.001 | [2.91, 3.68] | 0.182 | .129 | .476 | [-0.13, 0.49] | 3.476 | .156 | <.001 | [3.10, 3.85] |
|  |  |  |  |  |  |  |  |  |  |  |  |  |
| Lessons completed | 6.013 | .152 | <.001 | [5.65, 6.38] | 0.262 | .121 | .091 | [-0.03, 0.55] | 6.275 | .146 | <.001 | [5.92, 6.63] |
|  |  |  |  |  |  |  |  |  |  |  |  |  |
| Essays reviewed | 2.134 | .202 | <.001 | [1.65, 2.62] | -1.357 | .161 | .001 | [-1.74, -0.97] | 0.777 | .195 | <.001 | [0.31, 1.24] |
|  |  |  |  |  |  |  |  |  |  |  |  |  |

Table S2

*Pairwise Comparisons of District Differences in Students’ Mean Usability, Usefulness, and Desirability Ratings*

|  | District A–B | | | | District B–C | | | | District A–C | | | |
| --- | --- | --- | --- | --- | --- | --- | --- | --- | --- | --- | --- | --- |
|  | Mean Difference | *SE* | *p* | 95% CI | Mean Difference | *SE* | *p* | 95% CI | Mean Difference | *SE* | *p* | 95% CI |
| Usability | 0.237 | .040 | <.001 | [0.142, 0.332] | -0.125 | .031 | <.001 | [-0.200, -0.050] | 0.112 | .039 | .013 | [0.018, 0.206] |
|  |  |  |  |  |  |  |  |  |  |  |  |  |
| Usefulness | 0.250 | .042 | <.001 | [0.148, 0.352] | -0.112 | .033 | .002 | [-0.192, -0.032] | 0.138 | .042 | .003 | [0.037, 0.239] |
|  |  |  |  |  |  |  |  |  |  |  |  |  |
| Desirability | 0.209 | .061 | .002 | [0.062, 0.357] | -0.353 | .048 | <.001 | [-0.469, -0.237] | -0.143 | .061 | .057 | [-0.290, 0.003] |

Table S3

*Regression Models Predicting Usability*

|  | Model With Fall Predictors | | | | |  | Model With Spring Predictors | | | | |
| --- | --- | --- | --- | --- | --- | --- | --- | --- | --- | --- | --- |
|  | B | *SE* | β | *t* | *p* |  | B | *SE* | β | *t* | *p* |
| Intercept | 1.090 | .122 |  | 8.960 | <.001 |  | .503 | .109 |  | 4.598 | <.001 |
| District A | .189 | .045 | .149 | 4.185 | <.001 |  | .206 | .041 | .159 | 5.050 | <.001 |
| District B | -.085 | .035 | -.085 | -2.427 | .015 |  | -.036 | .032 | -.036 | -1.126 | .260 |
| Teacher caring | .117 | .034 | .137 | 3.416 | <.001 |  | .077 | .027 | .109 | 2.799 | .005 |
| Classroom belonging | .032 | .034 | .039 | .953 | .341 |  | .023 | .030 | .030 | .750 | .453 |
| Female | .013 | .030 | .014 | .441 | .659 |  | .001 | .028 | .001 | .021 | .984 |
| SPED | -.033 | .056 | -.018 | -.590 | .555 |  | .012 | .050 | .007 | .230 | .818 |
| LEP | .250 | .095 | .078 | 2.642 | .008 |  | .189 | .085 | .061 | 2.217 | .027 |
| FRL | .087 | .032 | .085 | 2.669 | .008 |  | .116 | .030 | .115 | 3.904 | <.001 |
| Grade | .124 | .039 | .100 | 3.199 | .001 |  | .132 | .036 | .107 | 3.692 | <.001 |
| Self-efficacy | .001 | .001 | .050 | 1.372 | .170 |  | .004 | .001 | .160 | 4.871 | <.001 |
| Liking writing | .052 | .026 | .071 | 1.994 | .046 |  | .113 | .024 | .156 | 4.770 | <.001 |
| Recursive process beliefs | .084 | .031 | .086 | 2.700 | .007 |  | .193 | .026 | .211 | 7.358 | <.001 |
| Writing proficiency | -.007 | .004 | -.069 | -2.089 | .037 |  | -.007 | .003 | -.068 | -2.285 | .022 |
| *F*_(13, 1033)_ = 10.938, *p* < .001, *R*^2^ = .348, adj. *R*^2^ = .121 | | | | | |  | *F*_(13, 1050)_ = 24.065, *p* < .001, *R*^2^ = .479, adj. *R*^2^ = .230 | | | | |

Table S4

*Regression Models Predicting Usefulness*

|  | Model With Fall Predictors | | | | |  | Model With Spring Predictors | | | | |
| --- | --- | --- | --- | --- | --- | --- | --- | --- | --- | --- | --- |
|  | B | *SE* | β | *t* | *p* |  | B | *SE* | β | *t* | *p* |
| Intercept | 1.138 | .129 |  | 8.853 | <.001 |  | .491 | .114 |  | 4.293 | <.001 |
| District A | .256 | .048 | .191 | 5.370 | <.001 |  | .248 | .043 | .180 | 5.817 | <.001 |
| District B | -.051 | .037 | -.048 | -1.385 | .166 |  | -.007 | .034 | -.007 | -.217 | .828 |
| Teacher caring | .098 | .036 | .109 | 2.729 | .006 |  | .043 | .029 | .057 | 1.488 | .137 |
| Classroom belonging | .049 | .035 | .056 | 1.380 | .168 |  | .082 | .032 | .100 | 2.580 | .010 |
| Female | -.023 | .032 | -.022 | -.708 | .479 |  | -.037 | .029 | -.036 | -1.289 | .198 |
| SPED | -.048 | .059 | -.025 | -.826 | .409 |  | <.001 | .053 | .000 | .000 | 1.000 |
| LEP | .168 | .100 | .050 | 1.680 | .093 |  | .133 | .089 | .041 | 1.495 | .135 |
| FRL | .064 | .034 | .060 | 1.871 | .062 |  | .101 | .031 | .094 | 3.262 | .001 |
| Grade | .045 | .041 | .034 | 1.089 | .277 |  | .029 | .037 | .022 | .779 | .436 |
| Self-efficacy | .000 | .001 | -.019 | -.511 | .610 |  | .001 | .001 | .045 | 1.394 | .164 |
| Liking writing | .109 | .028 | .140 | 3.948 | <.001 |  | .167 | .025 | .216 | 6.736 | <.001 |
| Recursive process beliefs | .148 | .033 | .143 | 4.497 | <.001 |  | .291 | .027 | .299 | 10.629 | <.001 |
| Writing proficiency | -.011 | .004 | -.096 | -2.927 | .003 |  | -.009 | .003 | -.084 | -2.860 | .004 |
| *F*_(13, 1038)_ = 11.012, *p* < .001, *R*^2^ = .348, adj. *R*^2^ = .121 | | | | | |  | *F*_(13, 1052)_ = 27.603, *p* < .001, *R*^2^ = .504, adj. *R*^2^ = .254 | | | | |

Table S5

*Regression Models Predicting Desirability*

|  | Model With Fall Predictors | | | | |  | Model With Spring Predictors | | | | | |
| --- | --- | --- | --- | --- | --- | --- | --- | --- | --- | --- | --- | --- |
|  | B | *SE* | β | *t* | *p* |  | B | *SE* | β | *t* | *p* |  |
| Intercept | .868 | .190 |  | 4.581 | <.001 |  | .165 | .172 |  | .957 | .339 |  |
| District A | -.008 | .070 | -.004 | -.117 | .907 |  | .000 | .064 | .000 | -.006 | .995 |  |
| District B | -.262 | .055 | -.169 | -4.801 | <.001 |  | -.210 | .051 | -.135 | -4.147 | <.001 |  |
| Teacher caring | .113 | .053 | .086 | 2.128 | .034 |  | .076 | .043 | .069 | 1.763 | .078 |  |
| Classroom belonging | -.021 | .052 | -.016 | -.400 | .689 |  | .082 | .048 | .069 | 1.721 | .086 |  |
| Female | .005 | .047 | .003 | .098 | .922 |  | -.058 | .044 | -.038 | -1.331 | .183 |  |
| SPED | -.105 | .086 | -.037 | -1.214 | .225 |  | -.085 | .079 | -.031 | -1.075 | .283 |  |
| LEP | .242 | .148 | .049 | 1.640 | .101 |  | .230 | .134 | .048 | 1.712 | .087 |  |
| FRL | .115 | .051 | .073 | 2.276 | .023 |  | .148 | .047 | .094 | 3.159 | .002 |  |
| Grade | .091 | .060 | .047 | 1.506 | .132 |  | .104 | .056 | .055 | 1.860 | .063 |  |
| Self-efficacy | -.002 | .001 | -.054 | -1.465 | .143 |  | -.003 | .001 | -.063 | -1.912 | .056 |  |
| Liking writing | .183 | .041 | .161 | 4.498 | <.001 |  | .331 | .037 | .293 | 8.863 | <.001 |  |
| Recursive process beliefs | .180 | .048 | .119 | 3.713 | <.001 |  | .285 | .041 | .200 | 6.921 | <.001 |  |
| Writing proficiency | -.009 | .005 | -.053 | -1.586 | .113 |  | -.007 | .005 | -.043 | -1.419 | .156 |  |
| *F*_(13, 1038)_ = 9.313, *p* < .001, *R*^2^ = .323, adj. *R*^2^ = .104 | | | | | |  | *F*_(13, 1052)_ = 21.823, *p* < .001, *R*^2^ = .461, adj. *R*^2^ = .212 | | | | | |
